# Jellyfish distribution in space and time predicts leatherback sea turtle hot spots in the Northwest Atlantic

**DOI:** 10.1371/journal.pone.0232628

**Published:** 2020-05-14

**Authors:** Bethany Nordstrom, Michael C. James, Boris Worm

**Affiliations:** 1 Biology Department, Dalhousie University, Halifax, Nova Scotia, Canada; 2 School of Biological Sciences, University of Western Australia, Perth, Australia; 3 Population Ecology Division, Fisheries and Oceans Canada, Dartmouth, Nova Scotia, Canada; Department of Agriculture, Water and the Environment, AUSTRALIA

## Abstract

Leatherback sea turtles (*Dermochelys coriacea*) migrate to temperate Canadian Atlantic waters to feed on gelatinous zooplankton (‘jellyfish’) every summer. However, the spatio-temporal connection between predator foraging and prey-field dynamics has not been studied at the large scales over which these migratory animals occur. We use 8903 tows of groundfish survey jellyfish bycatch data between 2006–2017 to reveal spatial jellyfish hot spots, and matched these data to satellite-telemetry leatherback data over time and space. We found highly significant overlap of jellyfish and leatherback distribution on the Scotian Shelf (r = 0.89), moderately strong correlations of jellyfish and leatherback spatial hot spots in the Gulf of St. Lawrence (r = 0.59), and strong correlations in the Bay of Fundy (r = 0.74), which supports much lower jellyfish density. Over time, jellyfish bycatch data revealed a slight northward range shift in the Gulf of St. Lawrence, consistent with gradual warming of these waters. Two-stage generalized linear modelling corroborated that sea surface temperature, year, and region were significant predictors of jellyfish biomass, suggesting a climate signal on jellyfish distribution, which may shift leatherback critical feeding habitat over time. These findings are useful in predicting dynamic habitat use for endangered leatherback turtles, and can help to anticipate large-scale changes in their distribution in response to climate-related changes in prey availability.

## Introduction

Highly migratory marine species have broad geographic ranges which can expose them to a range of natural and anthropogenic threats [1]. Migratory movements often take place between predictable locations, such as breeding and feeding habitats, that meet different life-history requirements [2]. Such critical habitat areas need to be properly identified, managed, and conserved in order to maintain viable populations [3]. Environmental variability and change, however, can make conservation efforts more difficult, as it may shift the quality and distribution of these habitats [4,5]. Hence, understanding and predicting such dynamic changes is crucial to designing adaptive management measures.

In this study, we investigate the spatio-temporal relationship between highly migratory leatherback sea turtles (*Dermochelys coriacea*), and their scyphozoan jellyfish prey in their summer foraging habitat off Atlantic Canada. Northwest Atlantic leatherbacks are currently considered endangered by the International Union for the Conservation of Nature [6] and Canadian species at risk legislation [7], and the future recovery of this subpopulation depends on conservation efforts in Atlantic Canadian foraging habitats [8]. These waters host one of the largest seasonal foraging aggregations of leatherbacks in the Atlantic [9], foraging predominantly on the larger lion’s mane (*Cyanea capillata*) and to a lesser extent on smaller moon jellyfish (*Aurelia aurita*) [10–13]. When present in Atlantic Canadian foraging habitats, leatherbacks may procure 20 to 59% of their annual energy budget over a single season [3]. It is, therefore, evident that Canadian foraging habitat is critical to leatherback turtle resource acquisition, however, the spatial relationship between leatherback sea turtles and their jellyfish prey has not been explored there [13–15].

This study builds upon previous work demonstrating a seasonal correlation between the timing of annual jellyfish blooms in Canadian waters and the arrival of migrating leatherbacks [13]. Here we explore the dynamic nature of this relationship in space and time and consider if observed climate variability may be affecting this relationship, and the distribution of leatherback foraging habitat. Changes in temperature can directly influence strobilation [16], growth rates [17], and medusa senescence [18, 19], as well as phytoplankton and zooplankton community structure [20, 21]. *C*. *capillata*, which is likely the most important forage species for leatherbacks in Atlantic Canada [11, 13, 22], is a cold-water species that could suffer from warming waters [16, 23], potentially shifting its distribution in response. The Northwest Atlantic has been warming since 1980, and is projected to continue on this trend faster than other ocean basins [24–26] making it a natural laboratory to study the complex effects of climate change on marine predators and their prey. Recent shifts in right whale habitat in the region, for example, were linked to climate-related shifts in copepod distribution, and resulted in large additional whale mortality due to emerging new threats in their novel feeding habitat [5, 27–29]. Such observations partly motivate the current study, as changes in leatherback scyphozoan prey distributions could potentially enhance threats to this endangered species.

We attempted to answer the following research questions: 1) How do spatial hot spots of jellyfish and leatherback sea turtles in Atlantic Canada compare, 2) are jellyfish ranges expanding or shifting over time, and 3) are any changes in jellyfish abundance and distribution driven by changes in sea surface temperature (SST)? Such knowledge could expand understanding of critical habitat for leatherbacks in Canadian waters, and inform effective recovery planning for this species.

## Materials and methods

### Study region

**Fig 1:**
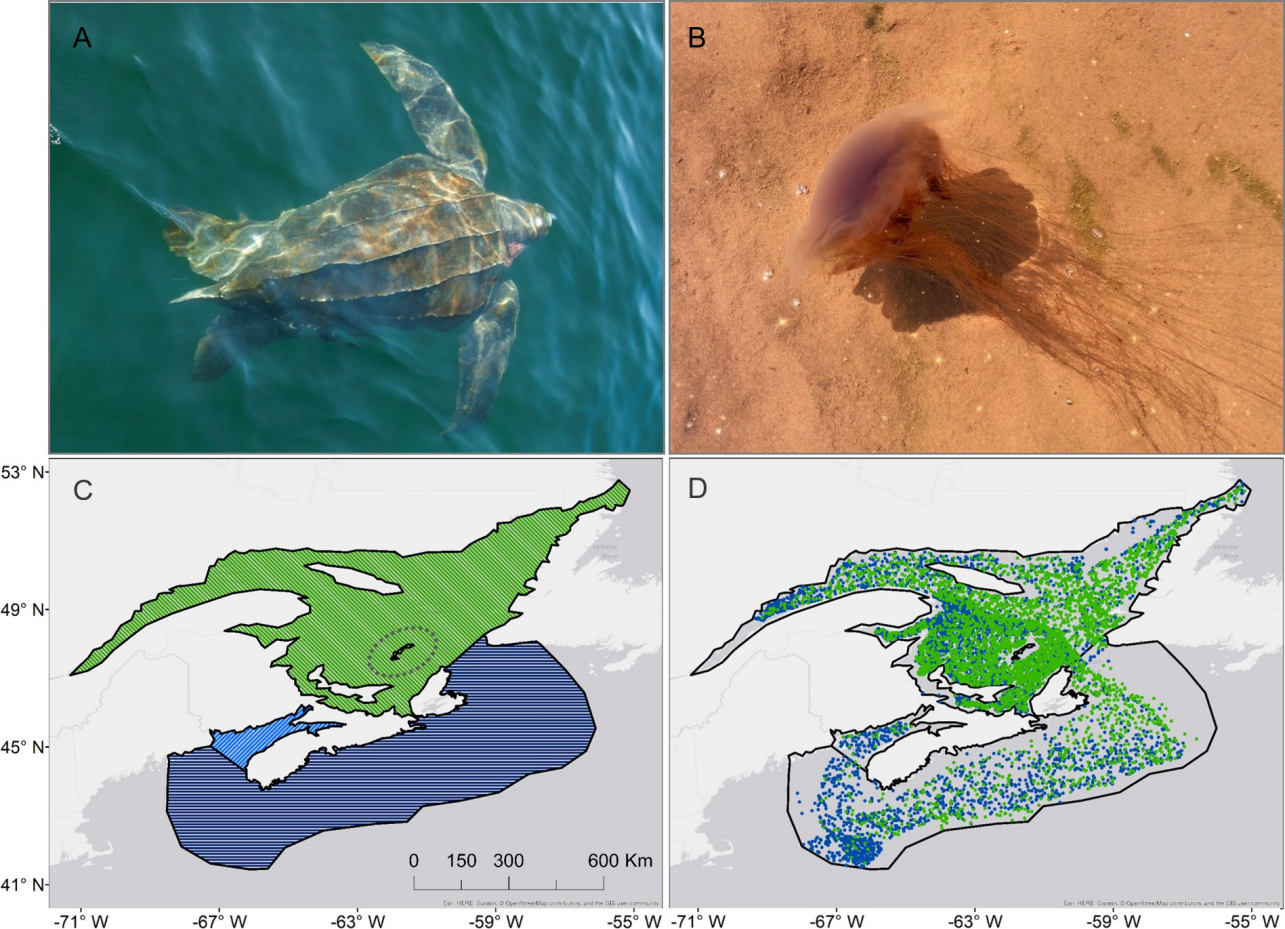
Study species and area in Atlantic Canada. A) Leatherback sea turtle (photo by Canadian Sea Turtle Network); B) lions mane jellyfish (*C*. *capillata*); C) biogeographical regions: Bay of Fundy in light blue, Scotian Shelf in dark blue, and the Gulf of St. Lawrence in green (Magdalen Islands circled within); regions are separated by black line; D) Department of Fisheries and Oceans trawl survey jellyfish presence (green dots) and absence (blue dots) 2006–2017.

Atlantic Canadian waters are known to be important seasonal foraging areas for leatherback sea turtles [9], which are found in three main areas: the Bay of Fundy, Scotian Shelf, and Gulf of St. Lawrence (Fig 1). These productive waters harbor seasonally abundant gelatinous zooplankton, including scyphozoan species such as lion’s mane and moon jellyfish [10–13]. Here we combined 12-year data sets for jellyfish and leatherback distribution throughout the region with sea surface temperature data to explore whether changes in ocean climate may affect jellyfish distribution and leatherback foraging habitat.

### Jellyfish data

The Canadian Department of Fisheries and Oceans (DFO) performs annual scientific trawl surveys to provide information on trends in biomass and abundance for commercially important fish species in the Maritimes Region [30, 31]. Although these are bottom trawls, mostly targeting groundfish species, all other species caught as bycatch, including jellyfish, are recorded.

Data were collected from the Bay of Fundy, Scotian Shelf, and Gulf of St. Lawrence regions (Fig 1). Jellyfish bycatch was recorded as ‘scyphozoan’ and not usually specific to species. Jellyfish were weighed to determine total wet weight (kg) for each trawl. Jellyfish data collection was implemented in annual trawl survey areas at different times: Scotian Shelf: 2006–2017, Southern Gulf of St. Lawrence: 1985–2017, and Northern Gulf of St. Lawrence: 2004–2017. Groundfish surveys on the Scotian Shelf occur for four to five weeks, normally centred on the month of July. The surveys in the Southern Gulf of St. Lawrence occur through the month of August (sentinel surveys, with observers on commercial fishing vessels) and the month of September (research vessel). The groundfish surveys in the Northern Gulf of St. Lawrence occur during the month of September. All of these groundfish surveys use a Western IIA trawl system, which has a cod-end mesh of 19mm, headline height of 3.5m and wingspread of 12m [32]. However, the research vessel and gear type may differ if there is required maintenance in a particular year. Outside of these bycatch data, there are currently no dedicated surveys to determine jellyfish distribution, abundance, or biomass in the Canadian Maritimes Region.

Survey data were rasterized into 0.5 x 0.5 degree gridded cells corresponding to the study region (Fig 1). Jellyfish wet weight was standardized into catch per unit of effort (CPUE) per month in each cell, by dividing by the total number of trawls in each cell. Jellyfish presence was also standardized into CPUE by dividing the number of trawls with jellyfish present, by the total number of trawls in each cell.

### Leatherback data

We considered binned density data from 62 leatherback turtles tracked in the study region (2006 to 2017) by DFO and collaborators using Argos satellite-linked tags. Specifically, we used daily interpolated median latitude and longitude positions of tagged turtles falling within 0.5 x 0.5 degree gridded cells corresponding to the Canadian Exclusive Economic Zone (turtle days per cell). Turtle days are here defined as the total number of days a leatherback turtle was present in each 0.5 x 0.5 degree cell. This cell binning approach captures the range of error associated with ARGOS location estimates and standardizes turtle location binning for satellite tags spanning multiple brands, models, and programmed transmission regimes.

### SST data

SST data from 2006 to 2017 were provided by DFO (Advanced Very High Resolution Radiometer [AVHRR] SST Dataset, Remote Sensing Group, Bedford Institute of Oceanography; Ocean Ecology Laboratory, Ocean Biology Processing Group—composites created by the Remote Sensing Group at BIO). Satellite-derived SST (AVHRR) geotiff files were downloaded in monthly intervals at 1.5 km resolution and converted to the monthly average in each 0.5 x 0.5 degree grid cell (executed in ArcGIS 10.5).

### Data analysis

#### Spatial overlap of jellyfish and leatherbacks

An optimized hot spot analysis was conducted based on the Getis-Ord Gi* statistic (executed in ArcGIS 10.5) to analyse spatial clustering of 1) jellyfish bycatch presence, 2) jellyfish bycatch biomass, and 3) leatherback turtle days. We used jellyfish bycatch presence to examine jellyfish distribution, and jellyfish bycatch biomass to examine jellyfish abundance. This optimized hot spot analysis tests the null hypothesis that the spatial relationship between neighbouring hot spots (high values) and cold spots (low values) is due to random clustering and is given as:

$$G_{i}^{*}=\frac{\sum_{j=1}^{n}w_{i,j}x_{j}-\bar{x}\;\sum_{j=1}^{n}w_{i,j}}{S\sqrt{\frac{n\sum_{j=1}^{n}w_{i,j}^{2}-{(\sum_{j=1}^{n}w_{i,j})}^{2}}{n-1}}}$$

where *x*_*j*_ is the attribute value for feature *j* (features are the value in each 0.5 x 0.5 degree cell for 1) standardized jellyfish presence, 2) standardized jellyfish biomass, and 3) leatherback turtle days), *w*_*i*,*j*_ is the spatial weight between the feature *i* and *j*, *n* is equal to the total number of features (the total number of 0.5 x 0.5 degree cells), and:

$$\bar{x}=\frac{\sum_{j=1}^{n}x_{j}}{n}$$

and:

$$S=\sqrt{\frac{\sum_{j=1}^{n}x_{j}^{2}}{n}}$$

Local patterns of standardized 1) jellyfish bycatch occurrence, 2) jellyfish weight, and 3) leatherback binned density, were each identified using a nearest-neighbour approach, and compared to the whole study area. The Getis-Ord Gi* statistics produces z-scores, which are represented numerically. Z-scores return a number that informs if the clustering of neighbouring points is attributed to random spatial processes (given their distance and value relative to the mean). A z-score greater than 1.65 represents a ‘hot spot’–a statistically significant spatial clustering of high positive values, whereas a z-score less than -1.65 represents a ‘cold spot’. No apparent clustering is indicated by a near zero z-score. Confidence levels are associated with z-score, where: z-score >2.58 = hot spot with 99% confidence; z-score 1.96 to 2.58 = hot spot with 95% confidence; z-score 1.65 to 1.96 = hot spot with 90% confidence; z-score (-1.65) to 1.65 = not significant; z-score (-1.65) to (-1.96) = cold spot with 90% confidence; z-score (-1.96) to (-2.58) = cold spot with 95% confidence; and z-score < (-2.58) = cold spot with 99% confidence. The hot spot analysis corrects for both multiple testing and spatial dependence using the False Discovery Rate [33].

Pearson’s correlation coefficient was calculated to determine whether there was significant overlap between jellyfish and leatherback hot spots, using the z-scores assigned to each cell. The z-scores of jellyfish presence and jellyfish weight were regressed against leatherback sea turtle z-scores in each of the three major regions (Bay of Fundy, Scotian Shelf, and Gulf of St. Lawrence–Fig 1).

#### Spatio-temporal trends of jellyfish bycatch in the groundfish surveys

To examine whether jellyfish distributions are shifting over time, the groundfish survey data were grouped into three bins, based on year: 2006–2009, 2010–2013, and 2014–2017. A hot spot analysis based on the Getis-Ord Gi* statistic (executed in ArcGIS 10.5) was used to analyse spatial clustering of both jellyfish presence and jellyfish weight in the groundfish surveys (see methods for ‘Spatial overlap of jellyfish and leatherbacks’ above for details).

To test whether jellyfish occurrence patterns are shifting over time due to the effects of changing temperatures, we calculated the average SST over the three months that the groundfish surveys take place (July, August, and September) for each region (Bay of Fundy, Scotian Shelf, and Gulf of St. Lawrence) for each year (2006 to 2017) (see S1 Table for spatial variations in mean SST). A linear regression was used to determine the rate of change in SST in each region over time. We also calculated the annual average latitude of jellyfish observations in the groundfish surveys in each region for each year. Pearson’s correlation coefficient was used to determine whether there was a strong relationship between the mean SST and mean average latitude of jellyfish observation per region over time.

#### Modelling trends of jellyfish biomass

The standardized jellyfish biomass data from the groundfish surveys were transformed using Tukey’s Ladder of Powers, as the data was not normally distributed and was heteroscedastic. All statistical modelling was done in R (version 3.6.0) [34]. To evaluate a potential relationship between SST and biomass of jellyfish in the groundfish surveys, a two-stage gamma (“hurdle”) model was fit to the data [35]. This generalized linear model (GLM) was chosen due to zero-inflation in our data, along with a continuous distribution (weight). The first stage modeled the probability of jellyfish presence compared to jellyfish absence using a binomial GLM and a logit link function. The second stage modeled the standardized jellyfish biomass data only, using a GLM with a gamma distribution and a log link function. Jellyfish presence was the dependent variable in stage 1 of the two-stage gamma model, while standardized jellyfish biomass was the dependent variable in stage 2 of the two-stage gamma model. Independent variables included: SST, year, region, latitude, longitude, and an interaction between SST and year. To account for multicollinearity, the variables SST and year were centered (subtract the mean method) before creating the interaction term. To ensure model assumptions were met, residuals were checked.

Model selection was performed by comparing all possible subsets of the full model using Akaike’s information criterion (AIC). Differences in AIC of <2 indicate there is not a substantial difference between models [36].

## Results

### Jellyfish data

**Fig 2:**
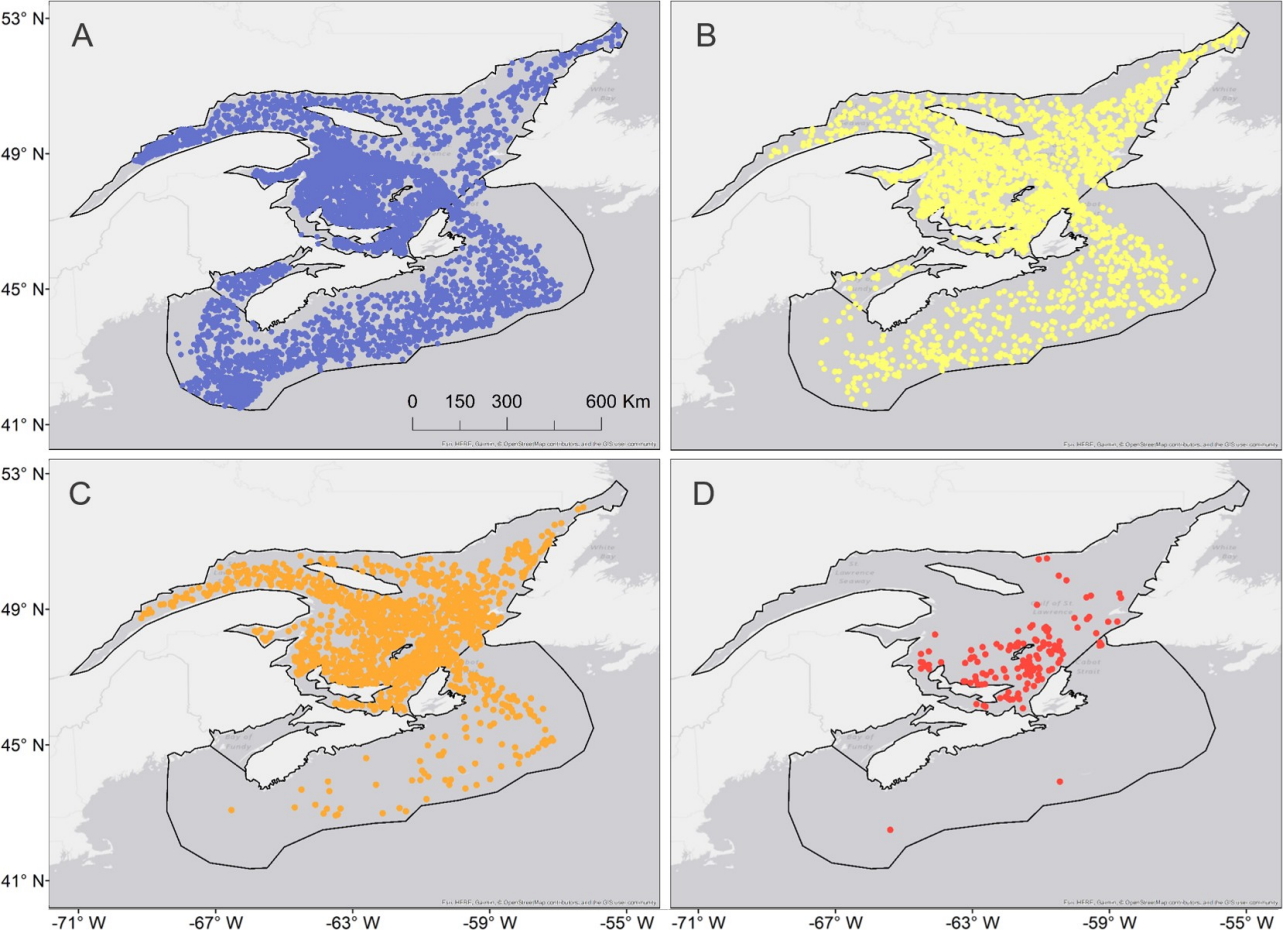
Jellyfish distribution by weight per survey tow. A) No jellyfish present in survey; B) Jellyfish present in survey, under 1kg (mostly likely between 1 to 5 individuals); C) Jellyfish present in survey, between 1 and 10kg biomass; and D) Jellyfish present in survey, over 10kg biomass.

A total of 8903 individual trawl survey tows were considered, with 117 locations in the Bay of Fundy, 2322 on the Scotian Shelf, and 6464 in the Gulf of St. Lawrence (Table 1). Jellyfish were present in 4137 trawls, with a total biomass of 9395.4 kg (Fig 1B). There were 2372 trawls with less than 1kg of jellyfish reported, spread quite evenly throughout the study region (Fig 2). Tows with catches over 10kg were mostly limited to the Gulf of St. Lawrence (Fig 2) (see S1 Fig for further breakdown of sampling effort).

**Table 1:** DFO groundfish surveys by region and year. ‘Biomass (kg)’ is the wet weight of jellyfish; ‘CPUE’ = catch per unit effort; ‘Jellyfish Pres’ is the number of trawls with jellyfish presence; and ‘% Presence’ is the percent of trawls with jellyfish presence.

	Bay of Fundy					Scotian Shelf					Gulf of St. Lawrence				
Year	Total Trawls	Biomass (kg)	CPUE (kg/tow)	Jellyfish Pres.	% Presence	Total Trawls	Biomass (kg)	CPUE (kg/tow)	Jellyfish Pres.	% JP	Total Trawls	Biomass (kg)	CPUE (kg/tow)	Jellyfish Pres.	% Presence
2006	0	0.00	0.000	0	0%	33	25.1	0.76	16	48%	580	1091.6	1.88	352	61%
2007	11	0.00	0.000	0	0%	173	32.8	0.19	29	17%	579	705.0	1.22	286	49%
2008	7	0.00	0.000	0	0%	160	26.6	0.17	51	32%	617	387.9	0.63	253	41%
2009	0	0.00	0.000	0	0%	207	28.5	0.14	44	21%	525	700.5	1.33	306	58%
2010	15	0.01	0.001	1	7%	224	44.0	0.20	59	26%	508	552.2	1.09	202	40%
2011	6	0.15	0.025	1	17%	246	133.1	0.54	74	30%	478	1030.9	2.16	325	68%
2012	7	0.00	0.000	0	0%	222	44.5	0.20	40	18%	529	1149.2	2.17	242	46%
2013	18	0.24	0.014	3	17%	279	201.0	0.72	111	40%	496	1321.5	2.66	291	59%
2014	14	0.56	0.040	1	7%	212	92.1	0.43	55	26%	623	538.7	0.87	334	54%
2015	15	1.64	0.109	3	20%	172	23.8	0.14	29	17%	640	456.3	0.71	394	62%
2016	10	0.00	0.000	0	0%	208	15.6	0.08	44	21%	560	570.2	1.02	327	58%
2017	14	1.57	0.112	7	50%	186	41.5	0.22	122	66%	329	178.4	0.54	135	41%
Total	117	4.16	0.036	16	14%	2322	708.7	0.31	674	29%	6464	8682.5	1.34	3447	53%
Mean	9.8	0.35	0.025	1	10%	193.5	59.06	0.32	56	30%	538.7	723.5	1.36	110	53%
	±5.9					±60.6					±84.2				

In the Scotian Shelf and Bay of Fundy datasets, 97.6% of jellyfish were identified simply as ‘scyphozoa’. The other 2.4% were assigned species codes of *C*. *capillata* and *Pelagia noctiluca*. In the northern Gulf of St. Lawrence dataset, 43.5% of jellyfish were identified as ‘scyphozoa’, and the remaining 56.5% were identified to species (26.1% *C*. *capillata*, 19.7% *Periphylla periphylla*, 8.9% *A*. *aurita*, 1.8% *Atolla wyvillei*).

### Spatial overlap of jellyfish and leatherbacks

**Fig 3:**
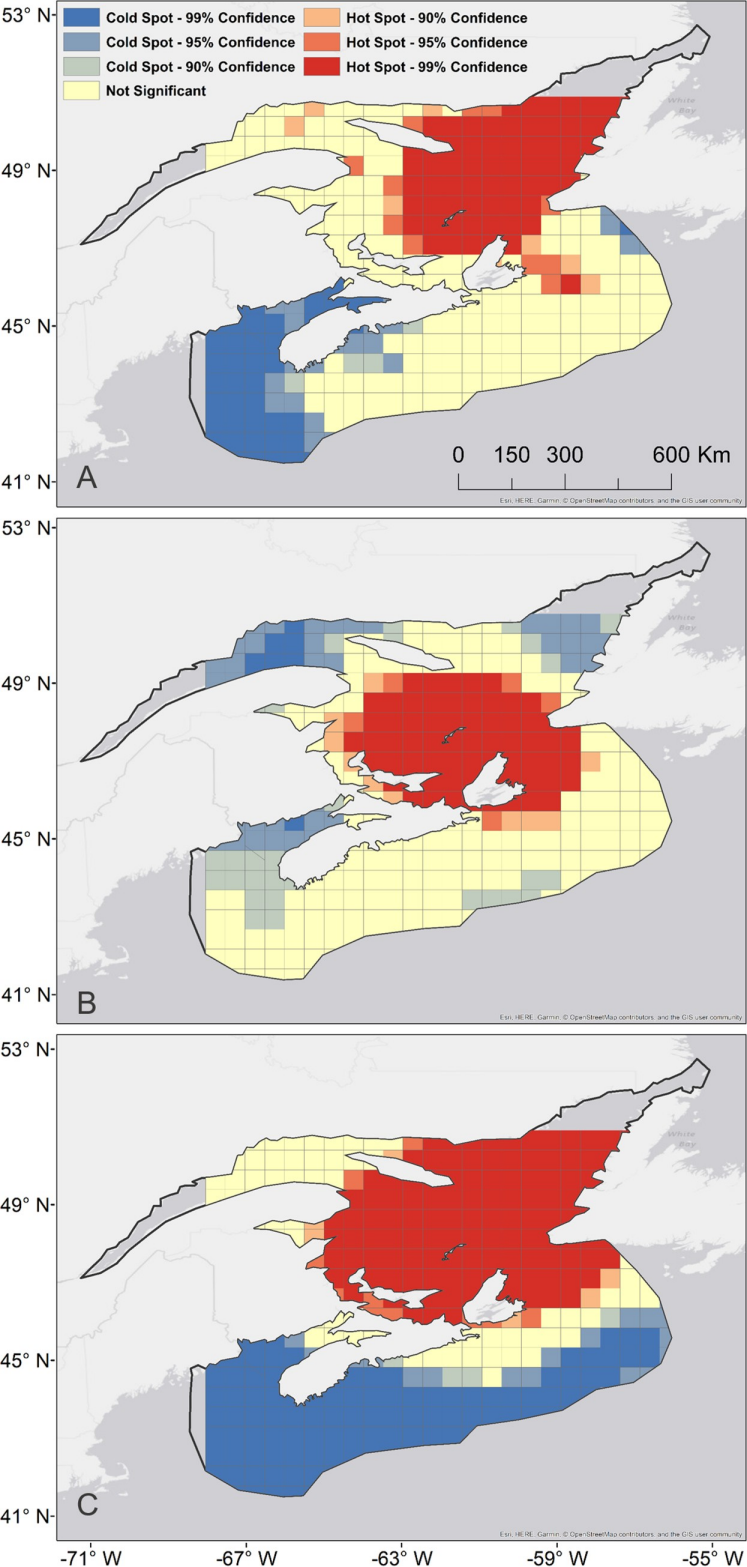
Optimized hot spot analysis (2006–2017). A) jellyfish presence (standardized), B) leatherback turtle days (days spent in each 0.5 x 0.5 degree cell), and C) jellyfish weight (standardized).

The optimized hot spot analysis highlighted somewhat congruent regional hot spots of 1) jellyfish presence, 2) jellyfish biomass, and 3) leatherback density, within the Gulf of St. Lawrence. These areas were classified as statistically significant spatial clusters, with 99% confidence (Fig 3).

The major jellyfish presence hot spot surrounds the Magdalen Islands in the Central Gulf of St. Lawrence, extending north along the western coast of Newfoundland and east to the Cabot Strait (Fig 3A). A smaller hot spot is seen off the northeast tip of Cape Breton. Cold spots are identified in the Bay of Fundy and south west Scotian Shelf (Fig 3A)

The statistically significant hot spot for leatherback binned density also surrounds the Magdalen Islands, extends through the Cabot Strait, and around the north east side of Cape Breton (Fig 3B). Significant cold spots are identified in the Bay of Fundy, the St. Lawrence Estuary, and the west coast of Newfoundland (Fig 3B).

The significant hot spot for jellyfish biomass covers most of the Gulf of St. Lawrence (Fig 3C). A statistically significant cold spot covers both the Bay of Fundy and the Scotian Shelf (Fig 3C).

**Fig 4:**
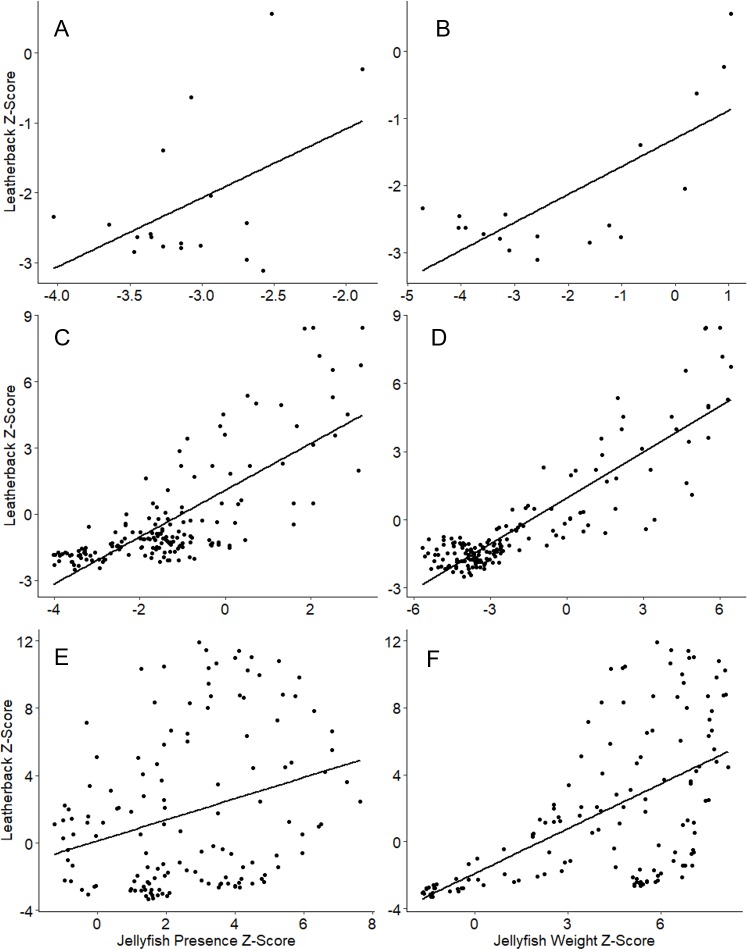
Predicting turtle habitat use from jellyfish data. Hot spot analysis z-scores compared for leatherbacks and jellyfish in each region with line of best fit. A) Bay of Fundy leatherback z-scores and jellyfish presence z-scores (r = 0.464, n = 18, p = 0.052); B) Bay of Fundy leatherback z-scores and jellyfish weight z-scores (r = 0.744, n = 18, p < 0.001); C) Scotian Shelf leatherback z-scores and jellyfish presence z-scores (r = 0.756, n = 166, p < 0.001); D) Scotian Shelf leatherback z-scores and jellyfish weight z-scores (r = 0.881, n = 166, p < 0.001); E) Gulf of St. Lawrence leatherback z-scores and jellyfish presence z-scores (r = 0.295, n = 137, p < 0.001); and F) Gulf of St. Lawrence leatherback z-scores and jellyfish weight z-scores (r = 0.588, n = 137, p < 0.001).

The z-scores assigned to each 0.5 x 0.5 degree cell for jellyfish presence or jellyfish biomass were each measured against the leatherback sea turtle z-scores in each of the three major regions (Bay of Fundy, Scotian Shelf, and Gulf of St. Lawrence) (Fig 4). Pearson’s correlation revealed a moderate association between jellyfish presence and leatherback turtle z-scores (r = 0.4640, n = 18, p = 0.0524), and a strong correlation between jellyfish weight and leatherback turtles z-scores (r = 0.7440, n = 18, p = 0.0004) in the Bay of Fundy (Fig 4A and 4B). Pearson’s correlation revealed a strong association between jellyfish presence and leatherback turtle z-scores (r = 0.7557, n = 166, p <0.001), and a very strong correlation between jellyfish weight and leatherback turtle z-scores (r = 0.8806, n = 166, p <0.001 on the Scotian Shelf (Fig 4C and 4D). Pearson’s correlation revealed a weak association between jellyfish presence and leatherback turtle z-scores (r = 0.2949, n = 137, p = 0.0005), and moderate association between jellyfish weight and leatherback turtle z-scores (r = 0.5881, n = 137, p<0.0001) in the Gulf of St. Lawrence (Fig 4E and 4F).

### Spatio-temporal trends of jellyfish bycatch

**Fig 5:**
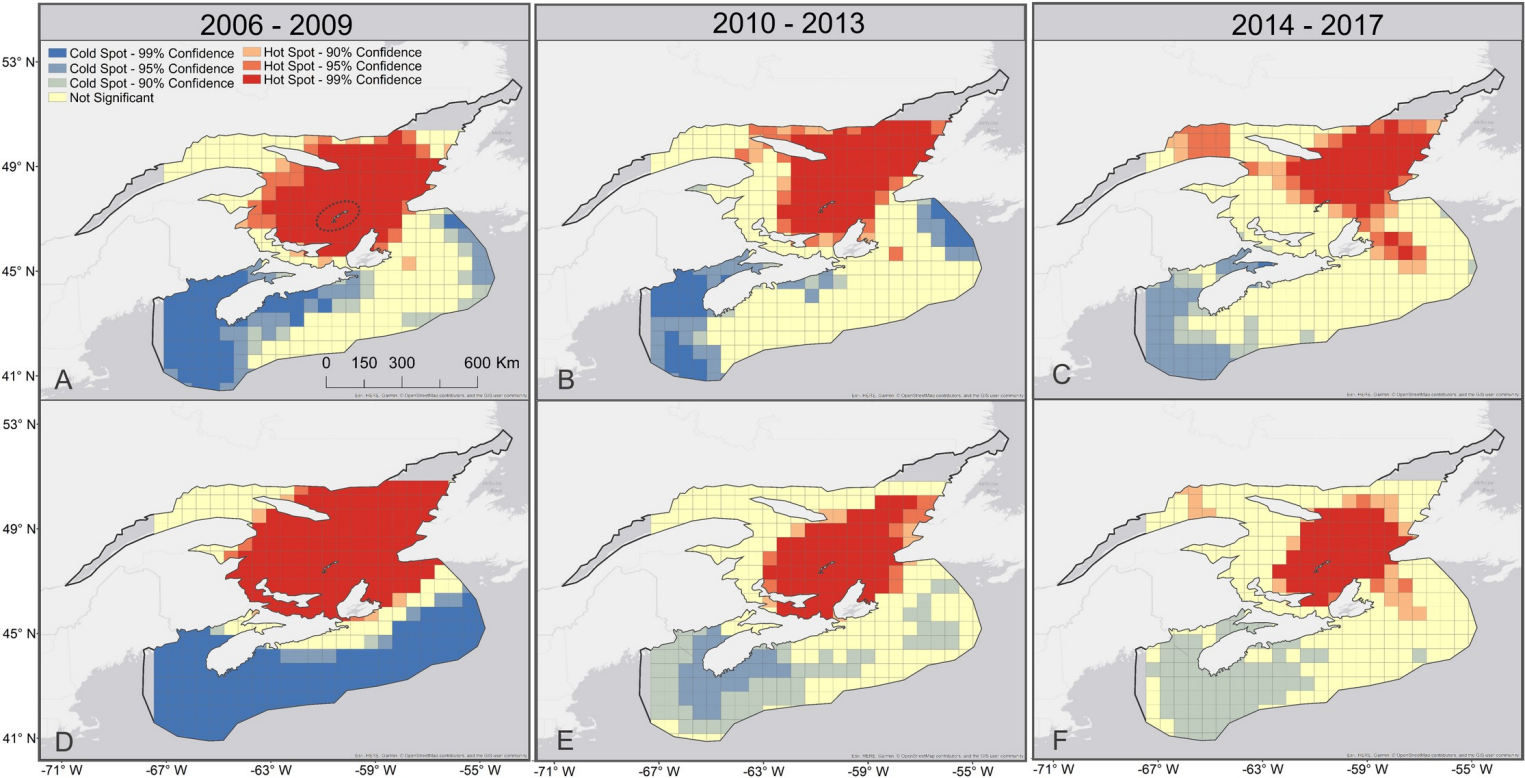
Optimized hot spot analysis of jellyfish occurrence (A—C), and jellyfish weight (kg) (D—F) in the groundfish surveys for 2006 to 2009 (A and D), 2010 to 2013 (B and E), and 2014 to 2017 (C and F). Magdalen Islands are circled within the Gulf of St. Lawrence (A).

The optimized hot spot analysis for jellyfish presence and weight over time highlighted hot spots within the Gulf of St. Lawrence, and in particular, the areas east and north of the Magdalen Islands as hot spots of jellyfish bycatch (both presence and weight) in the DFO surveys (Fig 5). These areas were classified as statistically significant spatial clusters, with 99% confidence.

Statistically significant clusters of 0.5 x 0.5 degree cells of jellyfish bycatch (standardized presence/absence and biomass) were identified for the periods 2006–2009, 2010–2013, and 2014–2017 (Fig 5; hot spots > 90% [z-scores > 1.65]). The clusters of jellyfish hot spots shift slightly between presence (Fig 5A–5C) and biomass data (Fig 5D–5F), and between each time period.

The center of the jellyfish hot spot distribution appeared to shift slightly northwards for both presence (Fig 5A–5C) and biomass data (Fig 5D–5F), but with regional variation, for example in the Cabot Strait area between Nova Scotia and Newfoundland. Statistically significant jellyfish cold spots are highlighted in similar regions for both presence (Fig 5A–5C) and biomass data (Fig 5D–5F), in the Bay of Fundy and around the south west Scotian Shelf.

**Fig 6:**
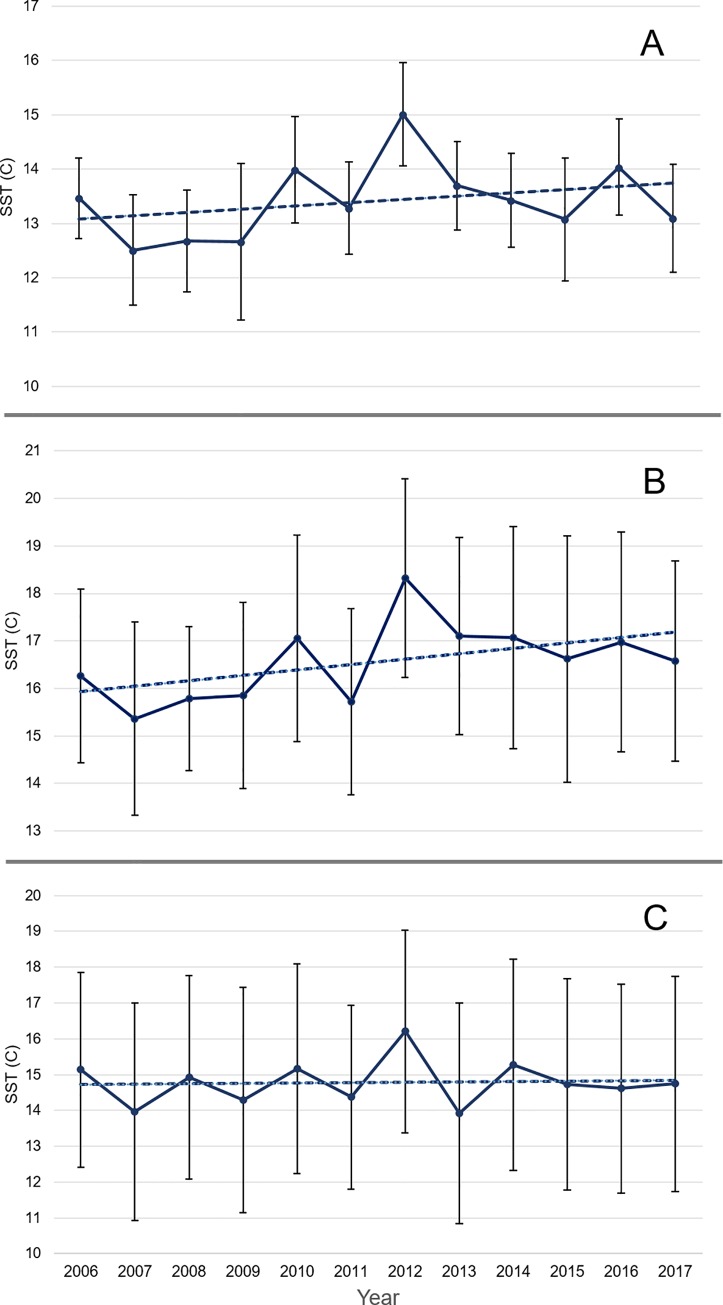
Mean SST over the study period (July, August, and September) from 2006 to 2017. A) Bay of Fundy SST over time, y = 0.0596x + 13.024; B) Scotian Shelf SST over time, y = 0.1144x + 15.814; and C) Gulf of St. Lawrence SST over time, y = 0.0111x + 14.707.

To test whether jellyfish occurrence patterns are shifting in response to changes in surface temperature, we calculated the average SST over the three month period that the surveys take place for each of the three regions for each year, and the annual average latitude of jellyfish observations in the groundfish surveys. The Bay of Fundy showed warming trends over the 12 year period, with an average increase of 0.06°C per year (Fig 6A). There was no discernible pattern of jellyfish distribution shifts, largely due to the sparseness of jellyfish in this region (i.e. 2006, 2007, 2008, 2009, 2012, and 2016). On the Scotian Shelf, SST showed a general warming trend over time, with an average increase of 0.11°C per year (Fig 6B). The average latitude of jellyfish observations showed a negative trend with a decrease of 0.091 degrees per year (S2 Fig). Pearson’s correlation revealed no linear correlation between the two (r = -0.1948, n = 12, p = 0.544) (S2 Fig). In the Gulf of St. Lawrence, there was a slight warming trend over time, with an increase of 0.01°C per year (Fig 6C). The average latitude of jellyfish observations showed a positive trend shifting northwards 0.056 degrees per year (S2 Fig). Pearson’s correlation revealed no linear association between SST and average latitude per year (r = 0.4377, n = 11, p = 0.1782) (S2 Fig).

### Modelling trends of jellyfish biomass

Among 15 candidate models (see S2 Table) testing for possible linkages between standardized jellyfish biomass in the groundfish surveys and explanatory variables, model glm11 was chosen based on AIC. It included the variables SST, year, region, latitude, longitude, and an interaction between SST and year (Table 2).

**Table 2:** Statistical analysis.

	*Presence model (Hurdle Stage 1)*				*Weight model (Hurdle Stage 2)*			
Variable	Coefficient	SE	z-value	P	Coefficient	SE	z-value	P
Intercept	-3.792	2.862	-1.325	0.185	-0.692	0.620	-1.116	0.265
SST	**0.358**	**0.046**	**7.783**	**p<0.001**	**0.077**	**0.009**	**8.282**	**p<0.001**
Year	**0.366**	**0.089**	**4.100**	**p<0.001**	**0.056**	**0.019**	**2.949**	**0.003**
GSL	**0.776**	**0.358**	**2.168**	**0.030**	0.177	0.110	1.615	0.106
SS	-0.061	0.342	-0.178	0.859	-0.076	0.107	-0.711	0.477
Latitude	**0.135**	**0.040**	**3.368**	**0.001**	0.005	0.008	0.590	0.555
Longitude	**0.129**	**0.019**	**6.845**	**p<0.001**	**0.016**	**0.004**	**3.629**	**p<0.001**
SST:Year	**-0.024**	**0.006**	**-4.298**	**p<0.001**	**-0.004**	**0.001**	**-3.627**	**p<0.001**

Results of two stage gamma hurdle models are shown. Stage 1 modelled the probability of the presence (non-zero) compared to the absence (0) of bycatch using a GLM with a binomial distribution and a logit link function. Stage 2 modelled the catch data only, using a GLM with a gamma distribution and a log link function. Significant variables (p < 0.05) in bold. GSL = Gulf of St. Lawrence; SS = Scotian Shelf.

GLMs indicated that both the probability of jellyfish presence (Hurdle model Stage 1) and the standardized weight (Hurdle Model Stage 2) significantly increased with increases in SST (p < 0.01, Table 2). Both jellyfish presence and standardized weight significantly increase over time (year, p < 0.01, Table 2). The regional effect was significant for the Gulf of St. Lawrence (p = 0.03, Table 2), while there were no significant effects for the Scotian Shelf or Bay of Fundy. There were no region effects on standardized weight (Stage 2, Table 2). The probability of jellyfish presence significantly increased with latitude (Stage 1, p < 0.01), and longitude (Stage 1, p < 001). There was no latitude effect on jellyfish weight, however, there was a significant positive effect of longitude on jellyfish weight (Stage 2, p < 0.01). Finally, there was a significant SST by year interaction for both jellyfish presence and standardized weight (Stage 1, Stage 2, both p < 0.01), indicating that the effects of SST on jellyfish slightly decreased over time.

## Discussion

The principal objectives of this study were to better understand the spatial overlap of jellyfish and leatherback sea turtles in Atlantic Canada, and to explore whether jellyfish distributions are changing with changing environmental conditions. The results provide insights into previously undocumented leatherback−jellyfish spatial interactions and contribute to the understanding of critical habitat for this endangered species. The results also highlight the utility of bycatch data as an ecological research tool in data-poor situations.

The optimized hot spot analysis highlighted congruent regional hot spots of 1) jellyfish presence, 2) jellyfish biomass, and 3) leatherback density, within the Gulf of St. Lawrence (Fig 3). The Gulf of St. Lawrence has been identified as important habitat for leatherback turtles [37]. Jellyfish distribution in Atlantic Canadian waters was examined using citizen science by Nordstrom et al. [13], where they found the Gulf of St. Lawrence had the highest records of jellyfish (in particular *C*. *capillata*), and CPUE of jellyfish per citizen scientist. The Gulf of St. Lawrence is described as a highly productive marine ecosystems with areas of upwelling [38, 39]. Jellyfish are often associated with areas of upwelling and other oceanographic features [40–42]. Phytoplankton (diatoms, dinoflagellates), zooplankton (copepods), and ichthyoplankton are seasonally abundant in the Gulf of St. Lawrence [43], and represent known food sources of scyphozoan jellyfish such as *C*. *capillata* and *A*. *aurita* [43]. The hot spots of jellyfish presence and biomass presented here are supported by previous literature that suggests jellyfish make up a large proportion of zooplankton biomass in the Gulf of St. Lawrence [44, 45].

Our analyses indicated that the Scotian Shelf was not statistically significant as a hot spot for jellyfish presence and satellite-tracked leatherback turtles (Fig 3A and 3B), or a statistically significant cold spot (Fig 3C). This is consistent with turtles moving through the Scotian Shelf en route to other foraging areas [46]. The hot spot analysis also highlighted comparable regional cold spots of 1) jellyfish presence, 2) jellyfish biomass, and 3) leatherback density, in the Bay of Fundy (Fig 3). Studies of leatherback distribution in Atlantic Canadian waters also found little to no leatherback presence in the Bay of Fundy, via volunteer sightings [9], and aerial surveys conducted for right whales [47, 48]. The Bay of Fundy was also not identified as important habitat for leatherback turtles based on movements of satellite-tagged leatherbacks [9]. A recent study on jellyfish phenology in Atlantic Canada revealed very few jellyfish observations in the Bay of Fundy [13], and the trawl surveys used in this study also corroborated these findings with only 14% of trawls having jellyfish present, and a CPUE of 0.036 kg per trawl (Table 1). The lack of turtles in the Bay of Fundy may be attributed to an inadequate prey field.

The hot spot analysis results for each region revealed moderate to strong correlations between standardized jellyfish biomass and leatherback binned density. Previous studies have suggested that leatherback migrations to and from Atlantic Canadian waters are driven by prey availability [3, 9, 14, 37, 49, 50], but this is the first time spatial distribution of predator and prey have been examined together. The leatherback hot spots were determined using binned density, or the number of days a leatherback was present in the 0.5 x 0.5 degree cell. Our results support the general behavioral ecology theory that predators will aggregate, and spend more time in areas of high prey density [51, 52]. The fidelity leatherback turtles show to foraging areas, and the amount of time spent in those regions indicate that turtles may be spending longer periods of time in areas of high jellyfish density [52–54]. A comparable study in the Irish Sea found that over a quarter of the variance in sighted leatherbacks (from citizen science) over a period of >50 years could be explained by coastal jellyfish hot spots [55]. This study shows, for the first time, overlapping areas of both high jellyfish and leatherback density in this important foraging area.

Both stages of two stage gamma modelling suggested that SST, year, longitude and the SST:year interaction were statistically significant predictors of jellyfish in the groundfish surveys (presence and biomass). SST had a positive influence on both jellyfish presence and biomass, suggesting that as temperature increases, so does the probability of jellyfish presence in a trawl, and of higher biomass. These findings are consistent with the hypothesis that increases in temperature will benefit many jellyfish species [23, 56]. Temperate scyphozoan jellyfish have a biphasic life cycle [57, 58]. After settling on the substrate, planula larva develop into sessile benthic polyps, which reproduce asexually, releasing free-swimming ephyrae. Ephyrae mature into pelagic medusae (the stage often referred to as a ‘jellyfish’), which reproduce sexually [57, 59, 60]. Release of ephyrae is often dictated by a specific thermal range [16, 23, 60–63], including *C*. *capillata* and *A*. *aurita*. Growth rates of ephyrae and medusae are also hypothesized to be influenced by temperature [14, 37].

Considering that the NW Atlantic is warming, and projected to keep warming at a faster rate than other ocean basins [24–26], we need to better understand how these changes will influence regional food-webs. Sherrill-Mix et al. [14] suggested that higher water temperatures may lead to increases in jellyfish growth and sexual maturity, ultimately accelerating the scyphozoan life cycle, resulting in earlier senescence. This could alter timing and duration of foraging opportunities available to leatherback sea turtles, such that there would be an earlier jellyfish signal, resulting in lower foraging opportunities later in the season [14] and, because of the sheer magnitude of the northward migration and constraints breeding and other life processes place on turtle departure times from low latitudes, an inability for some turtles to synchronize their arrival in Canadian waters with optimal resource availability. This idea is especially important to consider in Atlantic Canadian waters where one of the most common jellyfish species is *C*. *capillata* [13]. The average temperature in which deterioration of *C*. *capillata* medusae starts to occur is 19.1 ± 2.3°C (16.7°C to 23.3°C) in Connecticut waters [18]. Nordstrom et al. [13] found an increased probability of detecting *C*. *capillata* strandings (washed up on beaches) when SST reached 18°C along coastal Atlantic Canadian regions. SST reach values between 15 and 20°C in August in the Gulf of St. Lawrence [64], likely leading to widespread senescence of *C*. *capillata* medusae [13].

Another possible outcome of warming waters, is that geographical distributions of certain species may expand or contract [65, 66]. It is uncertain how *C*. *capillata*, a cold water jellyfish species, will be impacted by warming waters [16, 23, 60], and it is possible that their geographical range could shift north, modifying the current distribution of leatherbacks. In the eastern Bering Sea jellyfish populations have shifted northward in recent warming years [67]. The hot spot analysis results over time indicated that the jellyfish distribution (represented with presence/absence data) is slightly contracting to the north (Fig 5A–5C). This hot spot is mostly contained within the Gulf of St. Lawrence, and 26% of the jellyfish in Gulf of St. Lawrence groundfish surveys were identified as *C*. *capillata*. Recent years have seen warmer than usual annual water temperatures in the Atlantic Canadian waters, including 2014, 2015, 2016 [64, 68, 69]. We examined SST changes over the months in which groundfish surveys occurred (July, August and September). Within the Gulf of St. Lawrence, we did see a small increase in SST over the three month period per year (0.01°C), along with an increase in average jellyfish latitude observation (0.06 degree North). While the correlation between the two was not significant (most likely due to a small sample size), a moderate association was identified. While these results need to considered with caution, this may be indicative of a climate signal on jellyfish distribution within the Gulf of St. Lawrence. Ultimately, more research on NW Atlantic jellyfish, and perhaps specifically *C*. *capillata*, is needed to understand how changing climates may influence distributions.

Management of the endangered leatherback sea turtle relies on regular seasonal migrations, moving between foraging and breeding grounds. Seasonal distributions and density of migratory predators typically overlap with the peak abundance of their prey, maximizing seasonal prey availability [70, 71]. If there is a change in prey distributions, either spatially or temporally, predators will closely track these shifts [52, 72], which can become concerning if this brings the migratory predator into novel habitats and it becomes exposed to new anthropogenic and natural threats. For example, the critically endangered North Atlantic right whale has recently experienced shifts in feeding habitat, thought to be a result of rapid warming effects on copepods in the Gulf of Maine [29, 73]. Before this shift in habitat occurred, the National Oceanic and Atmospheric Administration (USA) and DFO had designated critical habitat based in their traditional feeding habitat, including the Gulf of Maine and southern Scotian Shelf [27, 74]. Right whale sightings in traditional feeding habitats began declining in 2012, and in 2015 an aggregation of right whales was discovered in the southern Gulf of St. Lawrence–an area outside of earlier-defined critical habitat [27–29]. This resulted in large additional whale mortality due to their vulnerability to marine traffic and entanglement threats in the new foraging habitat. The case of the North Atlantic right whale exemplifies how ecosystems and the species interactions within them are not static, and how anticipating change in these dynamic systems under climate change is needed. Understanding regional environmental drivers of jellyfish in Atlantic Canadian waters, and their sensitivity to projected climate change scenarios will be critical to future conservation of the leatherback sea turtle [3].

Longitude was a significant predictor of both jellyfish presence and jellyfish biomass, suggesting an increase from west to east. The hot spot over all years for jellyfish biomass (Fig 3C) encompasses the northeast area of our study area. Sherrill-Mix et al. [14] found longitude was a significant predictor of leatherback turtle departure from Atlantic Canadian waters, and that turtles remained in the region longer in waters around 63.6 degrees West. Our modelling results, and the findings of Sherrill-Mix et al. [14], may indicate that these areas provide better foraging opportunities for leatherback sea turtles [14]. The binomial GLM (stage 1) also suggested that latitude, and the regional effect for the Gulf of St. Lawrence were significant predictors of jellyfish presence in the groundfish surveys. If we interpret these results with the hot spot analysis, we do see the center of jellyfish biomass hot spots at slightly higher latitudes within the Gulf of St. Lawrence. While latitude and longitude were included in the model, spatial and temporal autocorrelation were not specifically addressed. Other factors which may influence jellyfish movement and distribution, such as currents and frontal systems [75, 76], were not considered here. These limitations should be considered when interpreting the effects of longitude and latitude on the model.

While the groundfish trawl surveys are not directly targeting jellyfish, they offer a consistent view of jellyfish occurrence in different regions across Atlantic Canadian waters. Jellyfish bycatch data present the opportunity to develop baseline knowledge on spatial jellyfish distributions in an area where no dedicated jellyfish surveys currently exist [77, 78]. The groundfish surveys in this study occur in three different regions: Scotian Shelf (including the Bay of Fundy), southern Gulf of St. Lawrence, and northern Gulf of St. Lawrence. While the surveys often employ the same research vessel and sampling gear, and sampling protocols are similar across regions, the regional surveys occur at different times. Scotian Shelf and Bay of Fundy surveys take place in the early part of the season (July), whereas the Gulf of St. Lawrence surveys occur in late summer (August and September). Potential sampling biases introduced by the lack of concurrent trawl surveys across all regions may affect interpretation of jellyfish catch data, and derived patterns of jellyfish distribution (e.g. southwest to northeast). Therefore, constraints such as different timing of sampling need to be carefully considered when combining regional datasets for spatial analysis purposes [79]. While different timing of the regional trawls in the present study may be a limitation, the jellyfish hot spots we identify are consistent with findings from citizen science jellyfish beach surveys [13], aerial survey observations of leatherback turtles [37] and leatherback turtle biotelemetry. For example, Nordstrom et al. [13] found *C*. *capillata* was the most common species recorded by citizen scientists in coastal Atlantic Canadian waters, with peak occurrence in the month of July, and spatial occurrence in the Gulf of St. Lawrence. This suggests that while surveys in each region do differ slightly in timing, the findings can be used to infer broad spatial patterns of jellyfish in Atlantic Canada. Fisheries bycatch data in other countries/jurisdictions has provided insights into spatio-temporal distributions of jellyfish as well [80–82].

Canada’s national endangered species legislation (Species at Risk Act) requires definition of critical habitat for listed species, including characterization of the functional attributes of that habitat. While leatherback turtle high use areas in Atlantic Canada have been described from shipboard and aerial sightings data [9, 37] and satellite telemetry data, importantly, until now (this study and [13]), quantification of the prey landscape in this region and its association with leatherback distributions had not been clarified. This represented a conspicuous gap in our understanding of this species’ biology, especially as leatherbacks migrate vast distances for the very purpose of foraging here, with fat stores acquired by turtles over a single foraging season supporting over 50% of their annual energy requirements [3]. While availability of corresponding jellyfish data (e.g. trawl survey data for the Newfoundland region were not available) restricted the spatial context of this study to a fraction of the areas sampled by satellite-tagged leatherback turtles in Canada, the present results indicate strong seasonal overlap in scyphozoan jellyfish and leatherback turtle hot spots in Atlantic Canada, and corroborate the importance of the Gulf of St. Lawrence, in particular, as an important foraging area for leatherbacks.

Seasonal foraging grounds, such as Atlantic Canada, should be of high conservation priority for the recovery of the endangered Northwest Atlantic leatherback subpopulation, as these areas offer reliable resources that enable turtles to acquire large energy stores in a relatively short time period. A primary threat to leatherback turtles in Atlantic Canadian waters is incidental entanglement in fishing gear [14, 83, 84]. The results presented here provide information that could be used to guide management actions for the endangered leatherback sea turtle. Protection of critical habitat for the leatherbacks in Canada should potentially extend beyond traditional static, or fixed area-based management, to include dynamic management approaches [3]. Dynamic management facilitates rapid responses to changes in the marine environment, through monitoring of near real-time data (biological, oceanographic, and economic data) [4]. Changing climate is likely to impact jellyfish distributions, and corresponding leatherback turtle foraging distributions in the temperate Northwest Atlantic. Therefore, to optimize leatherback management activities in space and time, we need to both understand and effectively monitor how environmental parameters influence leatherback prey distributions. This study suggests that recording of incidental catch of jellyfish in annual fishery surveys at broadly distributed sampling areas, and corresponding environmental data, is valuable to tracking the quality of leatherback habitat over time. We urge that these methods be expanded and combined with additional approaches to monitor and predict the distribution of important leatherback foraging habitat, and to mitigate anthropogenic threats to leatherbacks in these areas.

## Supporting information

S1 Fig Sampling effort in the study area for each time period (2006–2009, 2010–2013, and 2014–2017). A) total number of trawls in each 0.5 x 0.5 degree cell; B) jellyfish biomass (kg) in each 0.5 x 0.5 degree cell; C) catch per unit effort (CPUE) of jellyfish biomass (kg/tow) per 0.5 x 0.5 degree cell; D) jellyfish presence counts (number of trawls with jellyfish present) per 0.5 x 0.5 degree cell; and E) CPUE of jellyfish presence (jellyfish presence counts divided by the number of tows in each cell).
(TIF)

S2 Fig Average SST and average latitude per jellyfish observation. Average SST July, August, and September (blue line), and the average latitude per jellyfish observation (red line), for A) the Scotian Shelf, where r = -0.195 (B); and C) the Gulf of St. Lawrence, where r = 0.438 (D).
(TIF)

S1 Table Spatial variations in mean SST in the study area. ‘St. Dev’ = standard deviation, ‘CI (95%)’ = confidence interval (95%). Atlantic Canada is the entire study region (Bay of Fundy, Scotian Shelf and Gulf of St. Lawrence combined).
(TIF)

S2 Table Two stage hurdle model comparison. Model selection shown in bold. SST = sea surface temperature.
(TIF)

## References

[1] Harrison A-L, Costa DP, Winship AJ, Benson SR, Bograd SJ et al The political biogeography of migratory marine predators. Nat Ecol Evol. 2018; 2(10):1571–8. 10.1038/s41559-018-0646-8 30177802

[2] Lascelles B, Sciara GND, Agardy T, Cuttelod A, Eckert S et al Migratory marine species: their status, threats and conservation management needs. Aquat Conserv. 2014; 24(S2):111–27.

[3] Wallace BP, Zolkewitz M, James MC. Discrete, high-latitude foraging areas are important to energy budgets and population dynamics of migratory leatherback turtles. Sci Rep. 2018; 8:11017 10.1038/s41598-018-29106-1 30030495 PMC6054646

[4] Maxwell SM, Hazen EL, Lewison RL, Dunn DC, Bailey H et al Dynamic ocean management: Defining and conceptualizing real-time management of the ocean. Mar Policy. 2015; 58:42–50.

[5] Meyer-Gutbrod EL, Greene CH. Uncertain recovery of the North Atlantic right whale in a changing ocean. Glob Chang Biol. 2018; 24:455–464. 10.1111/gcb.13929 29084379

[6] The Northwest Atlantic Leatherback Working Group 2019. *Dermochelys coriacea* Northwest Atlantic Ocean subpopulation. The IUCN Red List of Threatened Species. 2019; e.T46967827A83327767. 10.2305/IUCN.UK.2019-2.RLTS.T46967827A83327767.en.

[7] COSEWIC (Committee on the Status of Endangered Wildlife in Canada). COSEWIC assessment and status report on the Leatherback Sea Turtle *Dermochelys Coriacea* in Canada. 2012; Committee on the Status of Endangered Wildlife in Canada, Ottawa.

[8] Tiwari M, Wallace BP, Girondot M. *Dermochelys coriacea* (Northwest Atlantic Ocean subpopulation). The IUCN Red List of Threatened Species. 2013; e.T46967827A46967830. 10.2305/IUCN.UK.2013-2.RLTS.T46967827A46967830.en.

[9] James MC, Sherrill-Mix SA, Martin K, Myers RA. Canadian waters provide critical foraging habitat for leatherback sea turtles. Biol Conserv. 2006; 133:347–357.

[10] Sipos JC, Ackman RG. Jellyfish (*Cyanea capillata*) lipids: fatty acid composition. J Fish Res Board Can. 1968; 25:1561–1569.

[11] James MC, Herman TB. Feeding of *Dermochelys coriacea* on medusae in the Northwest Atlantic. Chelonian Conserv Biol. 2001; 4:202–205.

[12] Heaslip SG, Iverson SJ, Bowen WD, James MC. Jellyfish support high energy intake of Leatherback Sea Turtles (*Dermochelys coriacea*): video evidence from animal-borne cameras. PLoS ONE. 2012; 7:e33259 10.1371/journal.pone.0033259 22438906 PMC3306388

[13] Nordstrom B, James MC, Martin K, Worm B. Tracking jellyfish and leatherback sea turtle seasonality through citizen science observers. Mar Ecol Prog Ser. 2019; 620:15–32.

[14] Sherrill-Mix SA, James MC, Myers RA. Migration cues and timing in leatherback sea turtles. Behav Ecol. 2007; 19:231–236.

[15] Graham WM, Martin DL, Martin JC. In situ quantification and analysis of large jellyfish using a novel video profiler. Mar Ecol Prog Ser. 2003, 254:129–140.

[16] Lucas CH, Graham WM, Widmer C. Jellyfish life histories: role of polyps in forming and maintaining scyphomedusa populations. Adv Mar Biol. 2012; 63:133–196. 10.1016/B978-0-12-394282-1.00003-X 22877612

[17] Hansson L. Effect of temperature on growth rate of *Aurelia aurita* (Cnidaria, Scyphozoa) from Gullmarsfjorden, Sweden. Mar Ecol Prog Ser. 1997; 161:145–53.

[18] Brewer RH. The annual pattern of feeding, growth, and sexual reproduction in Cyanea (Cnidaria: Scyphozoa) in the Niantic River Estuary, Connecticut. Biol Bull. 1989; 176:272–281. 10.2307/1541985 29300558

[19] Pitt KA, Lucas CH. Jellyfish Blooms. Dordrecht: Springer Netherlands; 2014. Chapter 4, Bloom and Bust: Why do blooms of jellyfish collapse?; p. 79–105.

[20] Li WKW, Harrison WG. Propagation of an atmospheric climate signal to phytoplankton in a small marine basin. Limnol Oceanogr. 2008; 53(5):1734–1745.

[21] Johnson C, Casault B, Head E, Spry J. 2016 Optical, chemical, and biological oceanographic conditions on the Scotian Shelf and in the Eastern Gulf of Maine in 2014. DFO Can Sci Advis Sec Res Doc. 2016; 2016/003:51.

[22] Wallace BP, Zolkewitz M, James MC. Fine-scale foraging ecology of leatherback turtles. Front Ecol Environ. 2015; 3:1–15.

[23] Holst S. Effects of climate warming on strobilation and ephyra production of North Sea scyphozoan jellyfish. Hydrobiologia. 2012; 690:127–140.

[24] Barnett TP, Pierce DW, Schnur R. Detection of anthropogenic climate change in the world’s oceans. Science. 2001; 292:270–274. 10.1126/science.1058304 11303099

[25] Lee SK, Park W, van Sebille E, Baringer MO, Wang C et al What caused the significant increase in Atlantic Ocean heat content since the mid-20th century? Geophys Res Lett. 2011; 38:L17607 10.1029/2011GL048856.

[26] Saba VS, Griffies SM, Anderson WG, Winton M, Alexander MA et al Enhanced warming of the Northwest Atlantic Ocean under climate change. J Geophys Res Oceans. 2016; 121(1):118–32. 10.1002/2015JC011346.

[27] Meyer-Gutbrod EL, Greene CH, Davies TA. 2018. Marine species range shifts necessitate advanced policy planning: The case of the North Atlantic right whale. Oceanography. 2018; 31(2):19–23, 10.5670/oceanog.2018.209.

[28] Davies KTA, Brillant SW. Mass human-caused mortality spurs federal action to protect endangered North Atlantic right whales in Canada. Mar Policy. 2019; 104:157–162.

[29] Record N, Runge J, Pendleton D, Balch W, Davies K et al Rapid climate-driven circulation changes threaten conservation of endangered North Atlantic Right Whales. Oceanography. 2019; 32(2).

[30] Fisheries and Oceans Canada. 2015 Maritimes research vessel survey trends on the Scotian Shelf and Bay of Fundy. DFO Can Sci Advis Sec Sci Response. 2016; 2016/011.

[31] Fisheries and Oceans Canada. Updated indices of abundance to 2015 for stocks of six groundfish species assessed by DFO Gulf Region. DFO Can Sci Advis Sec Sci Response. 2016; 2016/016.

[32] Carrothers PJG. Scotia-Fundy groundfish survey trawls. Can Tech Rep Fish Aquat Sci, no. 1609. Fisheries and Oceans Canada. 1988.

[33] Getis A, Ord JK. The analysis of spatial association by use of distance statistics. Geography. 1992; 24(3):198–206

[34] R Core Team. R: a language and environment for statistical computing. R Foundation for Statistical Computing, Vienna 2019 www.r-project.org

[35] Hurley I, Wringe BF, Heyer CED, Shackell NL, Lotze HK. Spatiotemporal bycatch analysis of the Atlantic halibut (*Hippoglossus hippoglossus*) longline fishery survey indicates hotspots for species of conservation concern. Conservation Science and Practice. 2019; 1(1).

[36] Burnham KP, Anderson DR. Model selection and multimodel inference: a practical information-theoretic approach, 2^nd^ edition New York: Springer; 2002.

[37] Mosnier A, Gosselin J-F, Lawson J, Plourde S, Lesage V. Predicting seasonal occurrence of leatherback turtles (*Dermochelys coriacea*) in eastern Canadian waters from turtle and ocean sunfish (Mola mola) sighting data and habitat characteristics. Can J Zool. 2019; 97(5):464–78.

[38] Gilbert M, Dufour. The Gulf of St. Lawrence marine ecosystem: an overview of its structure and dynamics, human pressures, and governance approaches. ICES CM. 2008; J:18.

[39] Devine L, Scarratt M, Plourde S, Galbraith PS, Michaud S et al Chemical and biological oceanographic conditions in the estuary and Gulf of St. Lawrence during 2015. DFO Can Sci Advis Sec Res Doc. 2017; 2017/034:48.

[40] Benson SR, Forney KA, Harvey JT, Carretta JV, Dutton PH. Abundance, distribution, and habitat of leatherback turtles (*Dermochelys coriacea*) off California, 1990–2003. Fish Bull. 2007; 105:337–347.

[41] Graham WM, Pagès F, Hammer WM. A physical context for gelatinous zooplankton aggregations: a review. Hydrobiologia. 2001; 451:199–212.

[42] Suchman CL, Daly EA, Keister JE, Peterson WT, Brodeur RD. Feeding patterns and predation potential of scyphomedusae in a highly productive upwelling region. Mar Ecol Prog Ser. 2008; 381:161–172.55. Dufour, R., Ouellet, P. Estuary and Gulf of St. Lawrence marine ecosystem overview and assessment report. Can Tech Rep Fish Aquat Sci, no. 2744E. Fisheries and Oceans Canada. 2007.

[43] Dufour R., Ouellet P. Estuary and Gulf of St. Lawrence marine ecosystem overview and assessment report. Can Tech Rep Fish Aquat Sci, no. 2744E. Fisheries and Oceans Canada. 2007.

[44] de Lafontaine Y, Demers S, Runge J. Pelagic food web interactions and productivity in the Gulf of St. Lawrence: a perspective, p. 99–123, In, Therriault JC (ed.) The Gulf of St. Lawrence: small ocean or big estuary? Can Spec Publ Fish Aquat Sci. 1991; 113.

[45] Locke A. The ichthyoplankton and invertebrate zooplankton of the coastal waters of Cape Breton Island: A review. Can Manuscr Rep Fish Aquat Sci no. 2606. Fisheries and Oceans Canada. 2002.

[46] Hamelin KM, Kelley DE, Taggart CT, James MC. Water mass characteristics and solar illumination influence leatherback turtle dive patterns at high latitudes. Ecosphere. 2014; 5:1–20.

[47] Brown MW, Tobin D. Vessel and aerial surveys for North Atlantic Right Whales in Canadian waters, 1998. 1999; Final Report: Contract F5245-8-0064, Bedford Institute of Oceanography, Halifax.

[48] Brown MW, Tobin D. Surveillance of North Atlantic Right Whales in Canadian waters: 1999. 2000; Final Report: Contracts F5245-9-0035, F5245-9-0193, Bedford Institute of Oceanography, Halifax.

[49] Houghton JDR, Doyle TK, Davenport J, Lilley MKS, Wilson RP et al Stranding events provide indirect insights into the seasonality and persistence of jellyfish medusae (Cnidaria: Scyphozoa). Hydrobiologia. 2007; 589:1–13.

[50] Gregr EJ, Gryba R, James MC, Brotz L, Thornton SJ. Information relevant to the identification of critical habitat for Leatherback Sea Turtles (*Dermochelys coriacea*) in Canadian Pacific waters. DFO Can Sci Advis Sec Sci Res Doc. 2015; 2015/079.

[51] Stephens DW, Krebs JR. Foraging theory. Princeton, NJ: Princeton University Press; 1987.

[52] Hays GC, Hobson VJ, Metcalfe JD, Righton D, Sims DW. Flexible foraging movements of leatherback turtles across the North Atlantic Ocean. Ecology. 2006; 87(10):2647–56. 10.1890/0012-9658(2006)87[2647:ffmolt]2.0.co;2 17089672

[53] James MC, Ottensmeyer AC, Myers RA. Identification of high-use habitat and threats to leatherback sea turtles in northern waters: new directions for conservation: Leatherback movements and conservation. Ecol Lett. 2005; 8:195–201.

[54] Bailey H, Benson SR, Shillinger GL, Bograd SJ, Dutton PH et al Identification of distinct movement patterns in Pacific leatherback turtle populations influenced by ocean conditions. Ecol Appl. 2012; 22:735–747. 10.1890/11-0633 22645807

[55] Houghton JDR, Doyle TK, Wilson MW, Davenport J, Hays GC. Jellyfish aggregations and leatherback turtle foraging patterns in a temperate coastal environment. Ecology. 2006; 87(8):1967–1972. 10.1890/0012-9658(2006)87[1967:jaaltf]2.0.co;2 16937635

[56] Purcell JE. Climate effects on formation of jellyfish and ctenophore blooms: a review. J Mar Biol Assoc UK. 2005; 85(3):461–476.

[57] Brotz L, Cheung WWL, Kleisner K, Pakhomov E, Pauly D. Increasing jellyfish populations: trends in Large Marine Ecosystems. Hydrobiologia. 2012; 690:3–20.

[58] Holstein TW, Laudet V. Life-history evolution: at the origins of metamorphosis. Curr Biol. 2014; 24:159–161. 10.1016/j.cub.2014.01.003 24556439

[59] Arai MN. A functional biology of Scyphozoa. London: Chapman & Hall; 1997.

[60] Gambill M, Peck MA. Respiration rates of the polyps of four jellyfish species: Potential thermal triggers and limits. J Exp Mar Biol Ecol. 2014; 459:17–22.

[61] Gröndahl F. A comparative ecological study on the scyphozoans *Aurelia aurita*, *Cyanea capillata* and *C*. *lamarckii* in the Gullmar Fjord, western Sweden, 1982 to 1986. Mar Biol. 1998; 97:541–550.

[62] Brewer RH, Feingold JS. The effect of temperature on the benthic stages of *Cyanea* (Cnidaria: Scyphozoa), and their seasonal distribution in the Niantic River estuary, Connecticut. J Exp Mar Biol Ecol. 1991; 152:49–60.

[63] Fuchs B, Wang W, Graspeuntner S, Li Y, Insua S, Herbst E-M, et al Regulation of polyp-to-jellyfish transition in *Aurelia aurita*. Curr Biol. 2014; 24(3):263–73. 10.1016/j.cub.2013.12.003 24440392

[64] Galbraith PS, Chassé J, Caverhill C, Nicot P et al Physical oceanographic conditions in the Gulf of St. Lawrence in 2015. DFO Can Sci Advis Sec Res Doc. 2016; 2016/056.

[65] Pinsky ML, Worm B, Fogarty MJ, Sarmiento JL, Levin SA. Marine taxa track local climate velocities. Science. 2013; 341:1239–1242. 10.1126/science.1239352 24031017

[66] Burrows MT, Schoeman DS, Richardson AJ, Molinos JG, Hoffman A et al Geographical limits to species-range shifts are suggested by climate velocity. Nature. 2014; 507:492–495. 10.1038/nature12976 24509712

[67] Stevenson DE, Lauth RR. Bottom trawl surveys in the northern Bering Sea indicate recent shifts in the distribution of marine species. Polar Biol. 2019; 42:407–421.

[68] Hebert D, Pettipas R, Brickman D, Dever M. Meteorological, sea ice and physical oceanographic conditions on the Scotian Shelf and in the Gulf of Maine during 2015. DFO Can Sci. Advis Sec Res Doc. 2016; 2016/083:49.

[69] Hebert D, Pettipas R, Brickman D, Dever M. Meteorological, Sea Ice and Physical Oceanographic Conditions on the Scotian Shelf and in the Gulf of Maine during 2016. DFO Can Sci Advis Sec Res Doc. 2018; 2018/016:53.

[70] Cushing DH. Plankton production and year-class strength in fish populations: an update of the match/mismatch hypothesis. Adv Mar Biol. 1990; 26:249–293.

[71] Visser F, Hartman K, Pierce G, Valavanis V, Huisman J. Timing of migratory baleen whales at the Azores in relation to the North Atlantic spring bloom. Mar Ecol Prog Ser. 2011; 440:267–279.

[72] Sims DW, Witt MJ, Richardson AJ, Southall EJ, Metcalfe JD. Encounter success of free-ranging marine predator movements across a dynamic prey landscape. Proc Biol Sci. 2006; 273(159):1195–1201. 16720391 10.1098/rspb.2005.3444 PMC1560279

[73] Pershing AJ, Alexander MA, Hernandez CM, Kerr LA, Bris AL et al Slow adaptation in the face of rapid warming leads to collapse of the Gulf of Maine cod fishery. Science. 2015; 350:809–812. 10.1126/science.aac9819 26516197

[74] Fisheries and Oceans Canada. Recovery strategy for the North Atlantic Right Whale (*Eubalaena glacialis*) in Atlantic Canadian Waters. Species at Risk Act Recovery Strategy Series. Fisheries and Oceans Canada, Ottawa. 2014; 68.

[75] Hay SJ, Hislop JRG, Shanks AM. North Sea scyphomedusae–summer distributions, estimated biomass and significance particularly for 0-group gadoid fish. Neth J Sea Res. 1990; 25:113–130.

[76] Doyle TK, Houghton JDR, Buckley SM, Hays GC, Davenport J. The broad-scale distribution of five jellyfish species across a temperate coastal environment. Hydrobiologia. 2007; 579:29–39.

[77] Bastian T, Stokes D, Kelleher JE, Hays GC, Davenport J, Doyle TK. Fisheries bycatch data provide insights into the distribution of the mauve stinger (Pelgia noctiluca) around Ireland. ICES J Mar Sci. 2011; 68:436–443.

[78] Aubert A, Antajan E, Lynam C, Pitois S, Pliru S, Vaz S, et al No more reason for ignoring gelatinous zooplankton in ecosystem assessment and marine management: Concrete cost-effective methodology during routine fishing trawl surveys. Mar Policy. 2018; 89:100–108.

[79] Chadwick EMP, Brodie W, Colbourne E, Clark D, Gascon D et al History of annual multi-species trawl surveys on the Atlantic Coast of Canada. Atlantic Zonal Monitoring Program Bulletin. 2007; 6:25–42.

[80] Aleksa K, Nero R, Wiggert J, Graham W. Descriptive density models of scyphozoan jellyfish in the northern Gulf of Mexico. Mar Ecol Prog Ser. 2018; 591:71–85.

[81] Decker M, Robinson K, Dorji S, Cieciel K, Barceló C et al Jellyfish and forage fish spatial overlap on the eastern Bering Sea shelf during periods of high and low jellyfish biomass. Mar Ecol Prog Ser. 2018; 591:57–69.

[82] Gawinski C, Huwer B, Munk P, Jaspers C. Biodiversity of gelatinous macrozooplankton: Quantitative assessment of data and distribution patterns in the southern and central North Sea during August 2018. Data in Brief. 2019; 25:104186 10.1016/j.dib.2019.104186 31388520 PMC6669316

[83] Hamelin KM, James MC, Ledwell W, Huntington J, Martin K. Incidental capture of leatherback sea turtles in fixed fishing gear off Atlantic Canada: incidental capture of leatherback turtles in Atlantic Canada. Aquat Conserv. 2017; 27(3):631–42. 10.1002/aqc.2733.

[84] Archibald DW, James MC. Evaluating inter-annual relative abundance of leatherback sea turtles in Atlantic Canada. Mar Ecol Prog Ser. 2016; 547:233–246. 10.3354/meps11648.

