# Supplementary figures and images for: Jellyfish distribution in space and time predicts leatherback sea turtle hot spots in the Northwest Atlantic
Source: PLoS One. 2020 May 14;15(5):e0232628. doi: 10.1371/journal.pone.0232628 (PMC7224493; doi:10.1371/journal.pone.0232628)

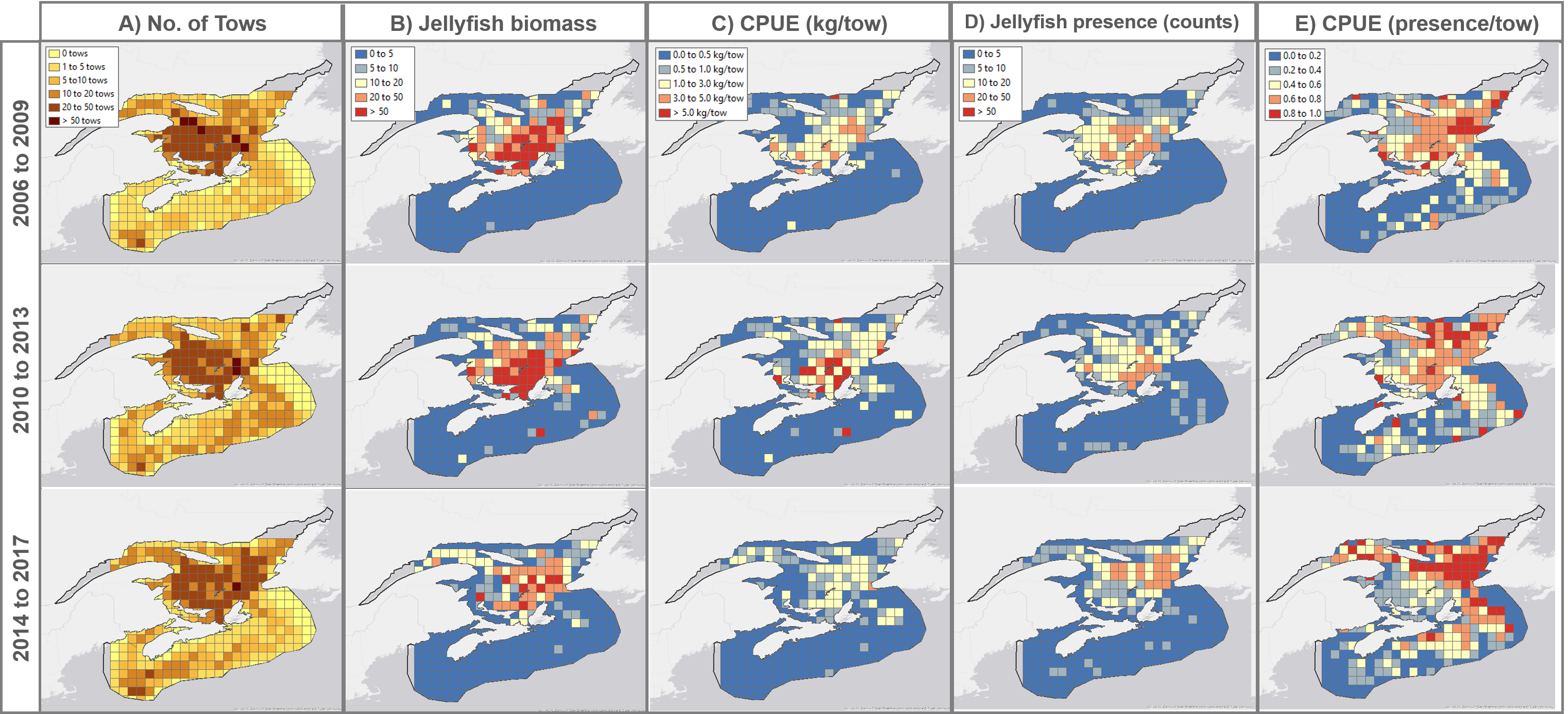

Supplement: S1 Fig — A) total number of trawls in each 0.5 x 0.5 degree cell; B) jellyfish biomass (kg) in each 0.5 x 0.5 degree cell; C) catch per unit effort (CPUE) of jellyfish biomass (kg/tow) per 0.5 x 0.5 degree cell; D) jellyfish presence counts (number of trawls with jellyfish present) per 0.5 x 0.5 degree cell; and E) CPUE of jellyfish presence (jellyfish presence counts divided by the number of tows in each cell). (TIF) [file pone.0232628.s001.tif]

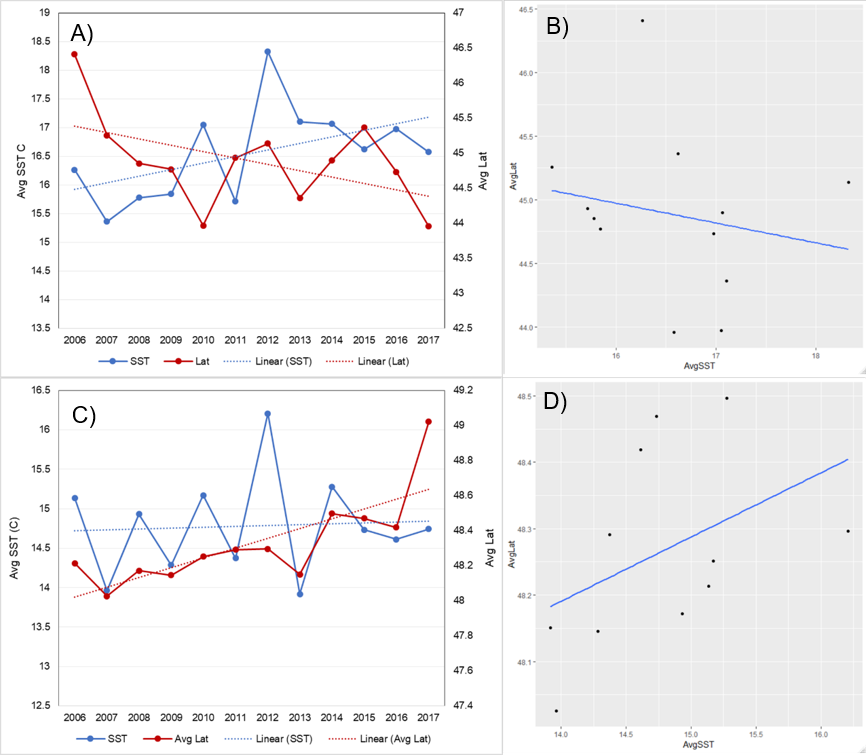

Supplement: S2 Fig — Average SST July, August, and September (blue line), and the average latitude per jellyfish observation (red line), for A) the Scotian Shelf, where r = -0.195 (B); and C) the Gulf of St. Lawrence, where r = 0.438 (D). (TIF) [file pone.0232628.s002.tif]

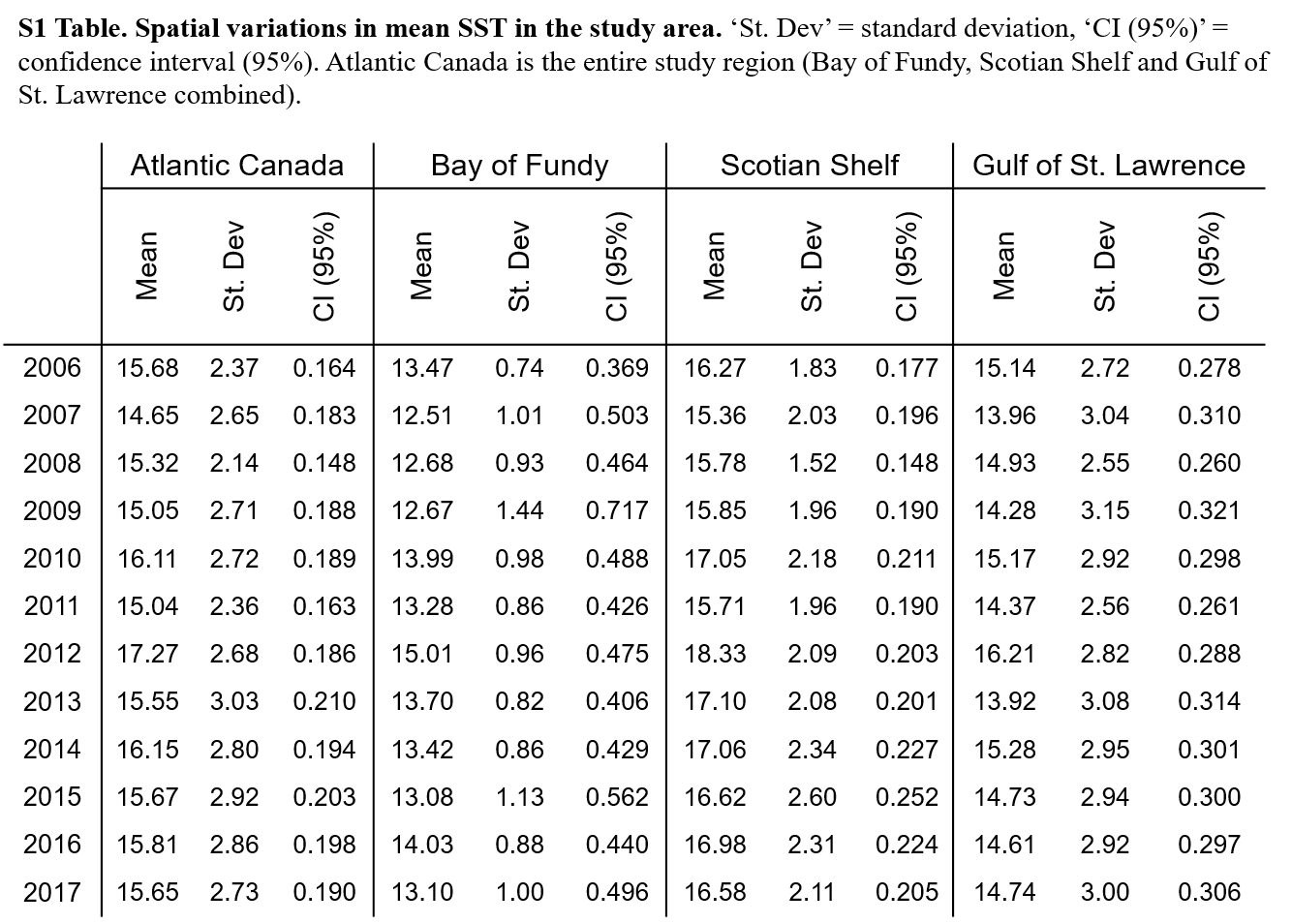

Supplement: S1 Table — ‘St. Dev’ = standard deviation, ‘CI (95%)’ = confidence interval (95%). Atlantic Canada is the entire study region (Bay of Fundy, Scotian Shelf and Gulf of St. Lawrence combined). (TIF) [file pone.0232628.s003.tif]

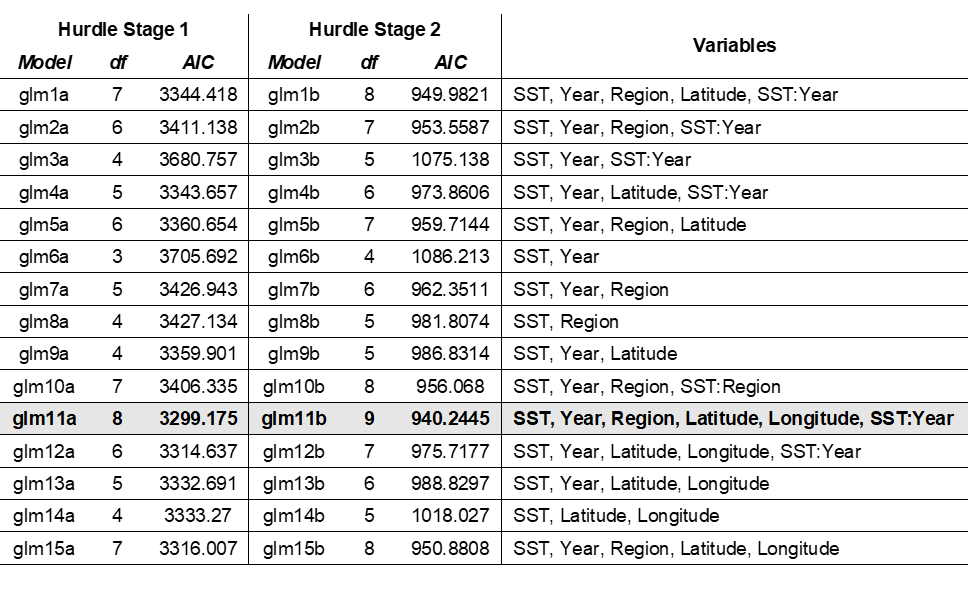

Supplement: S2 Table — Model selection shown in bold. SST = sea surface temperature. (TIF) [file pone.0232628.s004.tif]
